# Insect-Mediated Pollination of Strawberries in an Urban Environment

**DOI:** 10.3390/insects14110877

**Published:** 2023-11-14

**Authors:** Elsa Blareau, Pauline Sy, Karim Daoud, Fabrice Requier

**Affiliations:** 1 Université Paris-Saclay, CNRS, IRD, UMR Évolution, Génomes, Comportement et Écologie, 91198 Gif-sur-Yvette, France; 2 Institut d’Ecologie et des Sciences de l’Environnement de Paris, Sorbonne Université, 4 Place Jussieu, 75005 Paris, France; 3 LAB3S Sols Savoirs Saveurs, 32 Avenue Henri Varagnat, 93140 Bondy, France; 4 Laboratoire Régional du Suivi de la Faune Sauvage, 32 Avenue Henri Varagnat, 93140 Bondy, France

**Keywords:** biodiversity, crop pollination, ecosystem services, pollinator conservation, urban agriculture

## Abstract

**Simple Summary:**

Urban agriculture is a sustainable form of crop production for city-dwellers that requires insect pollinators to produce fruits and vegetables. However, few studies have tested whether urban pollinators are able to support the production of these urban crops. We carried out a study in an urban area near Paris (France) to test whether pollinators present in an urban environment contributed to the production of strawberries. From observational pollinator surveys, we found only wild pollinators visiting strawberry flowers, i.e., no honey bees were observed despite the presence of beehives nearby. We found that fruits were larger when pollinators could visit the flowers. Our results suggest that wild pollinators present in this urban environment are able to support strawberry production in an urban agricultural context.

**Abstract:**

Pollination services provided by a diversity of pollinators are critical in agriculture because they enhance the yield of many crops. However, few studies have assessed pollination services in urban agricultural systems. We performed flower–visitor observations and pollination experiments on strawberries (*Fragaria × ananassa*) in an urban area near Paris, France, in order to assess the effects of (i) insect-mediated pollination service and (ii) potential pollination deficit on fruit set, seed set, and fruit quality (size, weight, and malformation). Flower–visitor observations revealed that the pollinator community solely comprised unmanaged pollinators, despite the presence of beehives in the surrounding landscape. Based on the pollination experiments, we found that the pollination service mediated by wild insects improved the fruit size as a qualitative value of production, but not the fruit set. We also found no evidence of pollination deficit in our urban environment. These results suggest that the local community of wild urban pollinators is able to support strawberry crop production and thus plays an important role in providing high-quality, local, and sustainable crops in urban areas.

## 1. Introduction

Animal pollination is essential for the reproduction of 87.5% of all flowering plants [1] and contributes to yields of over half of the leading crops worldwide [2]. Crop pollination is valued at USD 195–387 billion annually [3]. Insects play a critical role in crop pollination [4,5,6], including, in particular, the Western honey bee *Apis mellifera*, which is known as an economically important pollinator in agriculture. Indeed, this species is managed by beekeepers who place beehives in close proximity to crops to ensure the pollination service is provided [7]. However, wild insect pollinators provide significant contributions to crop pollination in synergy with managed pollinators [8,9,10]. Since the 19th century, wild insect pollinators have suffered an important decline, and beekeepers have registered increased mortality rates in managed honey bee colonies [11,12,13]. Pressures on pollinator populations are due mainly to intensive agricultural practices, such as the use of pesticides and herbicides, and, in the case of wild pollinators, the loss of (semi-)natural areas for nesting habitats and wild flowers for foraging [11,12,13].

Beyond agricultural landscapes, pollinator populations also suffer from pressures in urban areas. On the one hand, the high proportion of impervious surfaces makes cities highly disturbed habitats, which favour generalist species and impact specialist species, leading to a biotic homogenisation of pollinator communities [14,15,16,17,18]. Nevertheless, urban landscapes could be considered a refuge for certain insect pollinators [19,20] given that they offer a high abundance of managed ornamental plants flowering over a longer period than unmanaged plant communities [21,22,23], and nesting habitats for cavity nesting bees are numerous [24]. These aspects of urban landscapes can allow cities to harbour diverse pollinator communities [25,26,27]. Despite a growing interest in urban pollinator diversity [28,29,30] and their pollination services [17,21,31,32], few studies assess whether these unique urban pollinator communities can provide sufficient pollination services to support urban crop production.

Urban food production, from allotments or private gardens, provides local and sustainable fruits and vegetables for the increasing human urban population, which represents over half of the world population [33]. A few recent studies have demonstrated the importance of taxonomically diverse pollinator communities, supported by florally diverse and dense gardens, for the urban production of various crops [34,35,36]. Indeed, pollinator species complement each other [5], and they can have synergistic effects on crop production, meaning that the service provided by the multi-species community is greater than the addition of the individual contributions of each species [6,37,38]. However, in urban landscapes, the diversity of pollinator communities may be affected by the rapid development of urban beekeeping [39,40] because managed honey bees, which are often numerically dominant, outcompete wild pollinators [41].

Here, we aim to evaluate the ability of an urban pollinator community to support strawberry production. This crop is commonly grown in allotments and urban gardens, and strawberry fruit quality has been shown to benefit from animal pollination in studies set in agricultural landscapes [2,42,43]. The cultivated strawberry (*Fragaria × ananassa*) has been bred since the 18th century [44], and it comprises numerous cultivars, which are produced by crossing genotypes with traits of interest [45], thus providing fruits that meet commercialisation criteria [46] and please consumers [47]. The contrast in pollinator community composition between urban and agricultural landscapes may affect the quality of the pollination service provided. However, little is known about the ability of urban pollinator communities to support strawberry fruit production as farmland pollinator communities do.

In the peri-urban environment of Paris (France), we surveyed the pollinator community visiting strawberry flowers and carried out pollination experiments. We used pollinator exclusion and hand pollination techniques to evaluate the effect of different pollination treatments on qualitative and quantitative measures of fruit production. These were the fruit set, fruit size, weight, malformations, and seed set. Fruit size and malformation are commercially important traits [46], whereas the seed set is closely linked to pollination. Indeed, the seeds, or achenes, are the true fruit of the strawberry [48]. Their formation is a direct result of ovule fertilisation, which requires pollination [48,49]. Although the fleshy part of the strawberry does not develop around unfertilised achenes, causing malformations to appear [48], its formation is less closely linked to pollination. We took a particular interest in evaluating the service provided by insect pollinators and the level of pollination deficit. This refers to a situation where the pollination service provided by insects present in the environment is not sufficient to maximise crop production [7,50]. We hypothesise that insect pollination increases the fruit set and fruit quality by increasing the fruit size, weight, and seed set and by decreasing fruit malformation. We further hypothesise that we will find a deficit in pollination such that pollen saturation (mediated by hand pollination) increases the fruit set, fruit size, and seed set and decreases occurrences of malformation compared to pollination by the local pollinator community. 

## 2. Materials and Methods

### 2.1. Study Site

This study was carried out on the campus of the French National Research Institute for Sustainable Development (IRD) and the neighbouring public park, located in Bondy, a town in the suburban environment of Paris, France (48.91° N 2.49° E). The site was located in a dense urban landscape with no crops or floral displays susceptible to attract pollinators away from our study site. It was the largest green area in a 4 km radius. The study area has a temperate climate (elevation: 57 m), the average yearly temperature is 11.6 °C, and the average annual rainfall is 723 mm [51] (Climate data, 2021). Mass flowering *Prunus* trees were present within the study site. One beehive of *Apis mellifera* was present in the study site, and three others were present in close proximity to the study site (Supplementary Information, Figure S1). To our knowledge, there were no other hives in close proximity to the study site (prospection performed in a radius of 500 m), but others could have been present in the honey bee foraging range. Indeed, data on hive presence are difficult to acquire in cities (e.g., the presence of apiaries on top of buildings or private gardens).

### 2.2. Biological Material

Forty strawberry (*Fragaria × ananassa*) plants of the variety “Deluxe” were bought from “Veni Verdi,” a local association, in February 2021. To our knowledge, this variety has never been used in a pollination study. The “Deluxe” strawberry variety is the product of a cross between two genotypes: the “Darselect” variety and genotype 16.01.18, selected in 2004 in France [52,53]. “Deluxe” is a short-day variety, meaning that it flowers in the spring when the day length is less than 12 h [52]. Although strawberries are relatively self-fertile, pollinator visits improve yields [2], particularly for the “Darselect” variety [54]. All plants were individually potted in 7.5 L pots in March 2021 with the same substrate, which consisted of compost and sand for drainage purposes. We added wood chippings above the soil of each pot to ensure moisture retention. Once potted, the plants were kept in the same location and, thus, the same climactic conditions until flowering began. When necessary, all plants were watered equal amounts within the same hour. 

### 2.3. Experimental Design

The forty strawberry plants were placed in the study area once flowering began on the 19 April 2021. The plants were randomly distributed among twenty locations (two plants per location, i.e., twenty spatial replicas) within the study site (Figure S1). Locations were assigned on QGIS [55] using a 60 × 60 m grid in which each section of the grid contained one or no plant locations (Figure S1). This grid helped us ensure that the plants were evenly spaced out across the study area. We avoided placing plants in areas of the study site that were accessible and commonly used by the public. All plants were well within the honey bee foraging range, which is, on average, 1.5 km [56]. The locations were, on average, 55.2 m from their nearest neighbouring location, with the minimum distance being 26.2 m and the maximum being 158.6 m. This design allowed us to encompass different microhabitats within the landscape in order to encapsulate the whole pollinator community present on the site, especially because wild pollinators have shorter foraging ranges than honey bees [40]. We considered flower visitors as a same community of pollinators due to the proximity of the locations instead of considering that locations have independent pollinator communities. We made sure that all location points were on grassy patches close to other floral resources where pollinators were more likely to be present. We placed two plants at each location to ensure we had enough flowers to perform flower observations and pollination treatments in all locations. 

### 2.4. Pollinator Observations and Pollination Treatments

Flower visitor observations were performed during the whole flowering period (from 20 April to 27 May 2021 (24 observation days)). Flower visitor observations consisted of 10 min observation sessions carried out between 9 a.m. and 6 p.m. We favoured days with temperatures above 12 °C with little cloud cover and no wind, although some observation sessions were carried out on cloudy days because several occurred during the flowering period. Each flower was observed several times (i.e., several 10 min observation sessions per flower), but never on the same day. Subsequent observations of the same flower were carried out at a later date at different times of the day in order to maximise our chances of seeing a diversity of pollinators, because different pollinators are active at different times of the day [5,57]. Over the flowering period, we carried out 30 h and 10 min of flower observations (181 time replicates of 10 min observations) on a total of 88 flowers (each observed, on average, 2.9 ± 1.1 times). We observed an average of 10.7 ± 7.4 flowers per day. An average of 4.4 ± 2.3 flowers were observed per location. Each flower visitor was counted and identified within the following 8 categories: honey bee (*Apis mellifera*), bumble bee (*Bombus* sp.), solitary bee, hoverfly (Syrphidae), other fly (Diptera), ant (Formicidae), thrips (Thysanoptera), or other insect. We chose this method over species identification because here we are interested in functional diversity, which is key for pollination services.

Independently of pollinator observations, pollination treatments were applied at each location during the same time period as pollinator observations to assess pollination services provided by the urban pollinator community. The four treatments were as follows: (i) flowers open to pollinator visits, (ii) flowers open to pollinator visits and cross-pollinated by hand, (iii) flowers excluded from pollinator visits (self or wind pollination only), and (iv) flowers cross-pollinated by hand and excluded from pollinator visits. By comparing self/wind and hand pollination, we measured pollinator dependence [50]. By comparing self/wind and open pollination, we measured pollination service, i.e., the contribution of insect pollinators to crop production [50]. By comparing open and hand pollination, we measured pollination deficit, i.e., whether pollinators are able to saturate the flower in pollen, thus allowing it to produce fruit at its highest potential [50]. By comparing open pollination with and without hand pollination, we measured whether insect pollination alone is sufficient to maximise fruit yield. The comparison of hand pollination and open pollination with hand pollination indicates whether hand pollination alone is enough to maximise fruit production, or whether an input from pollinators is necessary. Plants from the self/wind pollination treatment and the hand pollination treatment were bagged with mesh netting (Alt’Droso Maraichage, 0.8 × 0.8 mm mesh) to prevent pollinators from visiting these flowers (Figure S2). This method allowed for wind pollination, which can contribute to strawberry fruit set but does not maximise this measure [58]. For treatments that required hand pollination, pollen was collected from the study plants. We visited each flower twice within the same day with a paintbrush to ensure flowers of the same treatment received pollen from several other plants. A total of 172 flowers were selected for the pollination experiment, and 48 flowers were assigned to the open pollination treatment (2.3 ± 1.2 per location), 28 flowers were assigned to the hand and open pollination treatment (1.4 ± 0.9 per location), 63 flowers were assigned to the self/wind pollination treatment (3.2 ± 1.5 per location), and 35 flowers were assigned to the hand pollination treatment (1.8 ± 1.3 per location). All locations had the four different treatments.

### 2.5. Fruit Set and Fruit Quality

Once flowering was over, we measured the fruit set by recording whether each flower from each pollination treatment successfully produced a fruit or not. Fruits were then harvested once they were fully formed (i.e., as soon as the fruits had fully reddened) between 31 May and 10 June 2021. We recorded fruit malformation by considering a fruit with a clear aggregation of unfertilised achenes as showing a malformation (Figure S3). We measured fruit weight (Ohaus, Adventurer, precision 0.01 g, capacity 3100 g) and fruit size as the maximum width at the widest point (France Métrologie, accuracy 1 mm, capacity 1600 mm) within one day of harvesting. We chose width as the measure of fruit size because it is used to determine the commercial class of fruits [46]. The seed set, or seed number, was then counted once all fruits had been cropped. For maximum precision, strawberry flesh was separated from the seeds before counting using a small-meshed sieve, which collected only the seeds. 

### 2.6. Data Analysis

The statistical analysis was performed in R (version 4.2.2, R Foundation for Statistical Computing, Vienna, Austria) [59]. The fruit set was measured on a binary scale where a flower producing a fruit was given a score of 1, whereas one that failed to produce a fruit was attributed a score of 0. This measure of fruit production is used in pollination studies for various crops, including strawberry [42,60]. We used a mixed model approach to carry out our statistics analyses, with location as a random factor in each model. These models were computed using the lme4 R-package [61]. The MuMin R-package [62] was used to test the goodness of fit of mixed models, and the ggeffects R-package [63] was used to generate predicted values. A Generalized Linear Mixed Model (GLMM) with binomial error structure was used to test whether the fruit set (response variable) differed between pollination treatments (fixed factor). Fruit malformation was also recorded on a binary scale where a fruit showing a malformation received a score of 1, whereas a fruit showing no malformations received a score of 0. A binomial GLMM was used to test whether fruit malformation (response variable) was affected by fruit size and pollination treatments (fixed factors). A Linear Mixed Model (LMM) was used to test whether fruit size (response variable) was affected by fruit weight and pollination treatments (fixed factors). For this last model, we tested whether the size–weight relationship was non-linear using an assumption of quadratic pattern, because quadratic relationships between fruit quality measures have been reported for other crops [64,65]. Similarly, we used an LMM to test whether fruit size (response variable) was affected by seed set and pollination treatments (fixed factors). We also tested for a non-linear (quadratic) relationship between the fruit size and the seed set. If it was not significantly different from zero, we removed the quadratic term from the models. For all of the models (GLMMs and LMMs), we computed Type II Wald chi-squared tests to assess the effects of the explanatory variables using the car R-package [66], and we used Tukey’s post hoc tests to compare treatments using the multcomp R-package [67].

## 3. Results

### 3.1. Flower Visitors

**Figure 1:**
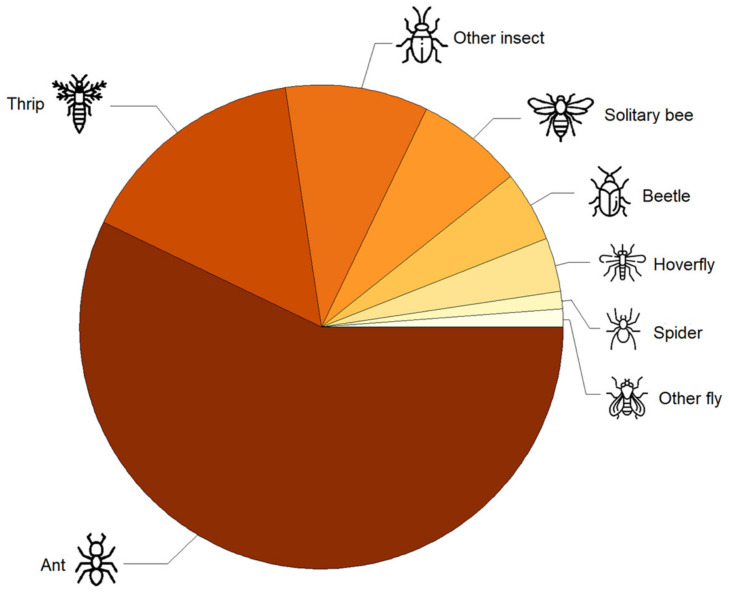
Diversity of strawberry flower visitors in an urban area. Icons from flaticon.com (accessed on 11 May 2023).

We recorded a total of 84 flower visitors over 257 single flower observation sessions. Among them, non-bee insects were dominant flower visitors, including ants (57%), followed by thrips (Thysanoptera) (17%) (Figure 1). Other flower visitors included solitary bees (7%), beetles (Coleoptera) (5%), hoverflies (Syrphidae) (4%), one spider (Aranea) (1%), and one other fly (Diptera) (1%) (Figure 1). Other insects made up 8% of the observations (Figure 1). No honey bees were observed visiting strawberry flowers despite the presence of four beehives in the surrounding landscape. 

### 3.2. Fruit Set

As part of the pollination experiment, a large majority of the 172 monitored flowers successfully reached fruit set (94.2%). Each plant successfully produced, on average, 5.2 ± 2.1 fruits, with 8.6 ± 3.3 fruits per location. We found no effect of pollination treatments on the fruit set (GLMM, *n* = 172, χ^2^ = 4.70, *p* = 0.195). Overall, 125 fruits were harvested from 32 different strawberry plants across all 20 location points. Issues with consumption by pests, or fruit picking by visitors, led to several fruits, for which fruit set success was recorded, not being included in the fruit quality measurements (22.8% of fruits). The following quality measurements were taken for the remaining 125 fruits. 

### 3.3. Fruit Malformation

**Figure 2:**
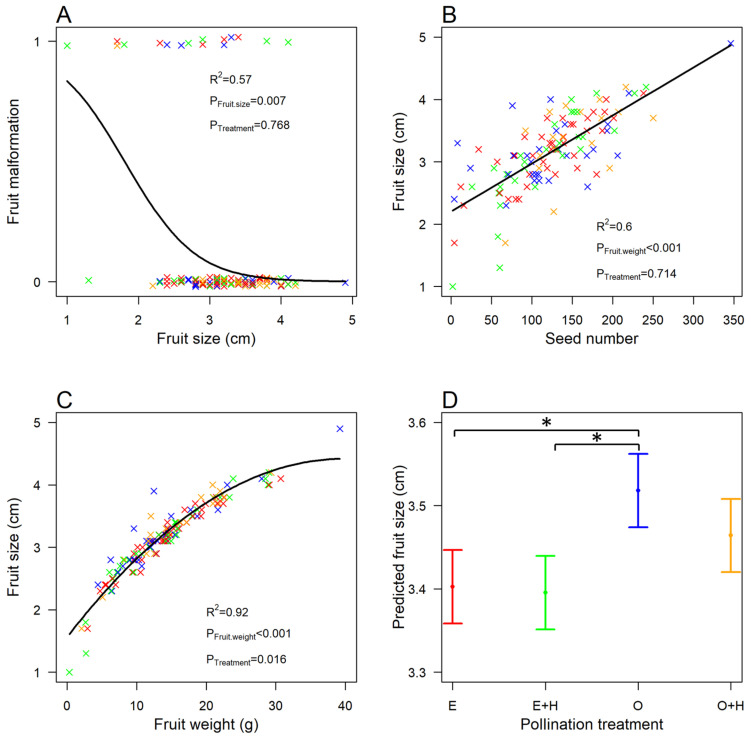
Relationship between (**A**) the probability of occurrence of a malformation and fruit size (maximum width measured in cm), (**B**) fruit size (maximum width in cm) and seed number, (**C**) fruit size and fruit weight (g), and (**D**) predicted fruit size values (mean ± se) and pollination treatment. Letters represent pollination treatments, with E for flowers excluded from pollinator visits (self or wind pollination only), E + H for flowers cross-pollinated by hand and excluded from pollinator visits, O for flowers open to pollinator visits, and O + H for flowers open to pollinator visits and cross-pollinated by hand. “×” signs represent the data, with colors representing the treatments (E in red, E + H in green, O in blue, and O + H in yellow). Thick lines show the GLMM and LMM predictions. Asterisk signs indicate significant differences between treatments (*p* < 0.05).

The majority of fruits showed no malformations (87.2%). We found that the probability of malformations decreased with fruit size (GLMM, *n* = 125, χ^2^ = 7.24, *p* = 0.007, Figure 2A), meaning that large fruits had a lower probability of malformation, but independently of the pollination treatment (*n* = 125, χ^2^ = 1.14, *p* = 0.768). In other words, insect-mediated pollination did not affect fruit malformation. 

### 3.4. Fruit Size

Pollination treatments significantly affected the fruit size–weight relationship (LMM, *n* = 125, χ^2^ = 10.28, *p* = 0.016, Figure 2C). Fruit size was greater for fruits from flowers open to pollinator visits (treatment O) than those from flowers excluded from pollinator visits (self or wind pollination only, treatment E) and flowers cross-pollinated by hand and excluded from pollinator visits (treatment E + H) (Table 1, Figure 2D). For all other pollination treatments, there was no difference in fruit size (Table 1, Figure 2D). We found an interesting, clear-cut, non-linear, quadratic relationship between fruit size and weight, which reached saturation for high values of weight (*n* = 125, χ^2^ = 1285.86, *p* < 0.001, Figure 2C). We also explored the effect of pollination on the relationship between fruit size and seed number, which showed fruit size increasing linearly with seed number (LMM, *n* = 125, χ^2^ = 142.36, *p* < 0.001, Figure 2B) but independently of the pollination treatment (*n* = 125, χ^2^ = 1.36, *p* = 0.714).

## 4. Discussion

Understanding how pollination affects crop production in urban landscapes is essential for the development of urban agriculture. Fruit quality improvement through animal-mediated pollination is well accepted [43,68,69,70,71], but little is known about the suitability of urban pollinator communities for urban crop production. We found that strawberry weight was higher for flowers exposed to insect pollination than for those excluded from it. This demonstrates that, despite the relative self-compatibility of strawberries, insect pollinators can support the production of strawberries in our local urban agricultural context. Another study found that insect pollination improves strawberry fruit weight in urban contexts when fruits were not damaged by pests [72]. Moreover, we found no increase in the fruit set or fruit quality from the treatment open to insect pollination compared to the pollen saturation treatments (i.e., flowers open to pollinator visits and cross-pollinated by hand), suggesting the absence of any pollination deficit. This suggests that there is sufficient pollen supply from insects present in the study site to maximise fruit quantity and quality. Because strawberries are not highly dependent on animal pollination [2], we can hypothesise that a small number of insect visits may be sufficient to saturate the flower in pollen. In addition, because our study crop has open flower morphology, it potentially attracts a range of generalist pollinators with short mouthparts [73]. This could be particularly true in urban areas, where generalist pollinators tend to be favoured [15,18]. In contrast, for wild plants, Bennett et al. (2020) [74] found that the pollination deficit was stronger in urban environments than in other types of landscapes. Former studies on strawberries have found varying results regarding pollination deficit. For instance, one study found effects of pollination deficit on fruit malformation [71], whereas another found no effects of pollination deficit on strawberry weight [75]. Overall, we want to emphasise that our results should be interpreted with care, because the sample size in this study was limited and this study was carried out during a single flowering season. Thus, we call for future studies to investigate the strawberry pollination services of insects in urban environments. If possible, future studies would benefit from including higher floral displays, i.e., more plants, and larger sample sizes.

We observed predominantly non-bee insects among our local urban pollinator community, which appeared to offer a positive contribution to urban strawberry production. In agricultural landscapes, studies find that the main strawberry flower visitors are frequently honey bees [20,60,70], although some studies find other pollinators as the main strawberry flower visitors, such as bumble bees [71,76], solitary bees [75], or flies [68]. Recent research has highlighted the importance of non-bee pollinators for crop production [10,77]. These results are promising for urban agriculture and emphasise the need for conservation of wild pollinators in urban landscapes. In the present study, the spatial design aimed to capture the conditions across the whole study site, but thus decreased the floral display available to attract pollinators. 

Recent studies suggest that urban areas could host more diverse pollinator communities than agricultural land because floral resources in cities are diverse [17,78]. Accordingly, we recorded only wild pollinators visiting strawberry flowers, even though four honey bee colonies were present in the landscape surrounding the study site, suggesting that managed pollinators did not intervene in urban strawberry pollination services. This result was unexpected, because honey bees are known to visit strawberry flowers in agricultural landscapes [43,60]. The ability of honey bees to forage at large distances from their hive [40,56,79] and to focus on massive floral resources through a strategy of “flower constancy” [80] could explain part of this result. Indeed, our strawberry plants were spread over 20 locations in our study area, offering a relatively sparse floral resource that could be less attractive to pollinators than other floral resources. In particular, honey bees could have favoured mass flowering cherry trees, which were present in the surroundings of the study area and overlapped in blooming time with our strawberries. Thus, the size of strawberry flower patches (i.e., floral display) could affect pollination services. With larger strawberry flower patches, we may assume a higher attraction of pollinators (in abundance and/or diversity), which could enhance pollination services (fruit set and fruit quality). On the other hand, with smaller strawberry flower patches, we may assume less pollinators (i.e., lower attraction to flower visitors), which could reduce pollination services. Such a hypothesis should be considered in future research. 

Instead of managed honey bees, we observed wild pollinators visiting strawberry flowers, such as ants, which are known to thrive in urban environments [81], and thrips, which have been previously noted as strawberry flower visitors [82]. We observed a relatively low abundance of flower visitors, possibly due to low floral display and non-optimal weather conditions during the sampling season. Solitary bees and hoverflies were also observed on strawberry flowers. These pollinators have much shorter foraging ranges than honey bees [40], suggesting that local, urban populations of wild pollinators provided the observed insect-mediated pollination services. This result supports claims of the importance of wild pollinators for urban crop production. For example, former studies have found similar results with jalapeño peppers [36], mango [83], cucumber, and eggplant [35]. 

Former studies have found that the strawberry fruit set increased when comparing flowers exposed to and excluded from insect visits [42,60,84]. Our results showed a generally high fruit set success rate (94.2%), indicating that the fruit set is independent of animal pollination for this variety. Fruit set being essential for crop producers, these crops have been bred to limit fruit set failures [85], which might explain why pollination has no effect on the fruit set. Moreover, although strawberry is referred to as modestly dependent on animal pollination for production [2], the intensity of this dependence is related to the crop variety [42,43], further explaining why the beneficial effects of insect pollination do not always translate onto the fruit set. 

Fruit size is a particularly important criteria for fruit commercialisation because large fruits (with a width greater than 25 mm) belong to a higher quality class and can be sold at higher prices [46]. Unsurprisingly, we found that fruit size was highly correlated with fruit weight. However, the saturation relationship observed suggests that fruit size is limited. This may be due to the fact that fruit shape is variable [86] and that for the same value of weight, some fruits may be wide and short whereas others may be long with a smaller width. Interestingly, there are no differences in fruit size between pollination treatments when considering the relationship between fruit size and seed set. Another study reported that the benefits of cross-pollination for strawberry weight (highly correlated with size) do not appear for all cultivars [87], indicating that cultivars may vary in their dependence on pollinators. Thus, the “Deluxe” cultivar used in our experiment could be one for which the benefits of insect pollination are not detectable on all measures of fruit quality. An increase in fruit size with a higher seed number is linked to a decrease in the fruit area with malformations because the fleshy part of the fruit develops around each seed, i.e., each fertilised achene [48]. Thus, flesh does not develop around unfertilised achenes, thereby producing small fruits with malformations. This metric is also important for strawberries’ economic value. We confirmed former results showing that very few fruits present any malformation [88], and that the pollination treatments were unrelated to fruit malformation [89]. However, other studies have found positive effects [54,69,90], including for var. Darselect, one of var. Deluxe’s parents [54], suggesting that effects on fruit malformation are variable. Because our exclusion method did not prevent wind pollination or crawling insects from visiting flowers, these may have affected pollination, with a potentially positive effect on fruit set and potentially reducing fruit malformation. Interestingly, another study found a low proportion of misshapen fruits in strawberries, with organic farming practices reducing the occurrences of malformations [49]. Indeed, we grew our strawberries organically because this agricultural practice is often used in urban agricultural systems. Reducing the occurrence of malformations is key for producers because the presence of malformations renders fruits unmarketable [46]. Thus, urban agricultural systems, which are key for sustainable development and local food security [46], associated with wild pollinator conservation could support producers’ economic outcomes [91]. 

During our pollinator observations, we noticed that the exclusion technique used did not prevent crawling insects, such as ants, from visiting strawberry flowers, despite this method being commonly used [43,68,69]. Similarly, another study reported issues with excluding small, crawling insects, such as thrips, using this exclusion method [92]. This generalised technical bias could lead to an underestimation of insect pollination and an overestimation of pollination deficit. Indeed, if small insects can get through nets and contribute to pollination in the self/wind and hand pollination treatments, then the observed benefit of insect pollination will be underestimated, and the observed pollination deficit will be overestimated. Nevertheless, we can assume a marginal effect of such crawling insects on pollination experiments given that ants are known as non-efficient pollinators in general [93]. Indeed, despite ant visits to flowers, we show the positive effects of flying insect visits to strawberry flowers on fruit size. Moreover, regarding hand pollination, our method for measuring pollination deficit involved hand pollination using a paintbrush. We found that fruits from the “hand pollination only” treatment were not significantly bigger than those from the self/wind pollination treatment. This result may be explained by the potential damage to flowers during flower handling and the use of a paintbrush. This methodological issue could prevent us from detecting effects of pollination deficit. These issues could be improved upon in future research to draw more robust conclusions on the benefits of insect-mediated pollination and the adverse effects of pollination deficit on urban crop production. 

## 5. Conclusion and Perspectives

Our results suggest that the urban community of wild pollinators supported strawberry fruit production, with mostly non-bee pollinators present on our study site. Our study is a first step towards understanding the role of urban wild pollinators on crop pollination services for strawberry. We call for future research to expand upon these results by studying urban pollination services at larger spatial scales on various crops in urban agricultural areas, particularly those known to highly depend on insect-mediated pollination. Such future research could corroborate our findings and strengthen our conclusions regarding the benefits of insect pollinators for urban crops, and this research would benefit from improved experimental design with spatial replication, larger sample sizes, and dense floral display. The findings may differ depending on urban policies regarding biodiversity conservation or the degree of urbanisation, because these parameters are known to affect pollinator community diversity [26] and could therefore have a knock-on effect on pollination services. Indeed, one study found that planting “bee-friendly” plants next to strawberry plants increased the yield and fruit quality in an urban area [94]. Urban conservation strategies to boost pollinator diversity, including sewing native wildflower strips, reducing pesticide use, or limiting mowing in urban green spaces [19,28], could promote pollinator diversity and the production of high-quality urban crops.

## Figures and Tables

**Table 1:** Tukey’s test values comparing fruit size for different pollination treatments from the GLMM testing the effects of fruit weight and pollination treatment on fruit size. Letters represent pollination treatments, with E for flowers excluded from pollinator visits (self or wind pollination only), E + H for flowers cross-pollinated by hand and excluded from pollinator visits, O for flowers open to pollinator visits, and O + H for flowers open to pollinator visits and cross-pollinated by hand. In bold are treatments that significantly differ in fruit size (*p* < 0.05).

Pollination Treatments	Estimate	Standard Error	*p* Value
E + H vs. E	−0.007	0.042	0.998
**O vs. E**	**0.116**	0.041	**0.027**
O + H vs. E	0.062	0.048	0.570
**O vs. E + H**	**0.122**	0.045	**0.033**
O + H vs. E + H	0.069	0.051	0.530
O + H vs. O	−0.054	0.050	0.405

## Data Availability

Data are publicly available through the figshare repository https://doi.org/10.6084/m9.figshare.24187542.v1, accessed on 8 November 2023 [95].

## Supplementary Materials

The following supporting information can be downloaded at: https://www.mdpi.com/article/10.3390/insects14110877/s1, Figure S1: Location of the study area and study sites, with apiary and plant locations; Figure S2: (a) Strawberry plants after potting, (b) example of two strawberry plants at their designated location with pollinator exclusion bags around the open flowers, (c) a strawberry flower excluded from insect visits by means of a pollinator exclusion bag, (d) a Diptera visiting a strawberry flower; Figure S3: Example of a strawberry from the experiment showing a malformation.
