# Supplementary material for: Insect-Mediated Pollination of Strawberries in an Urban Environment
Source: Insects. 2023 Nov 14;14(11):877. doi: 10.3390/insects14110877 (PMC10671972; doi:10.3390/insects14110877)

Insect-mediated pollination of strawberries in an urban environment

Elsa Blareau, Pauline Sy, Karim Daoud, Fabrice Requier

Supplementary Information

Figure S1. Location of the study area and study sites with apiary and plant locations.

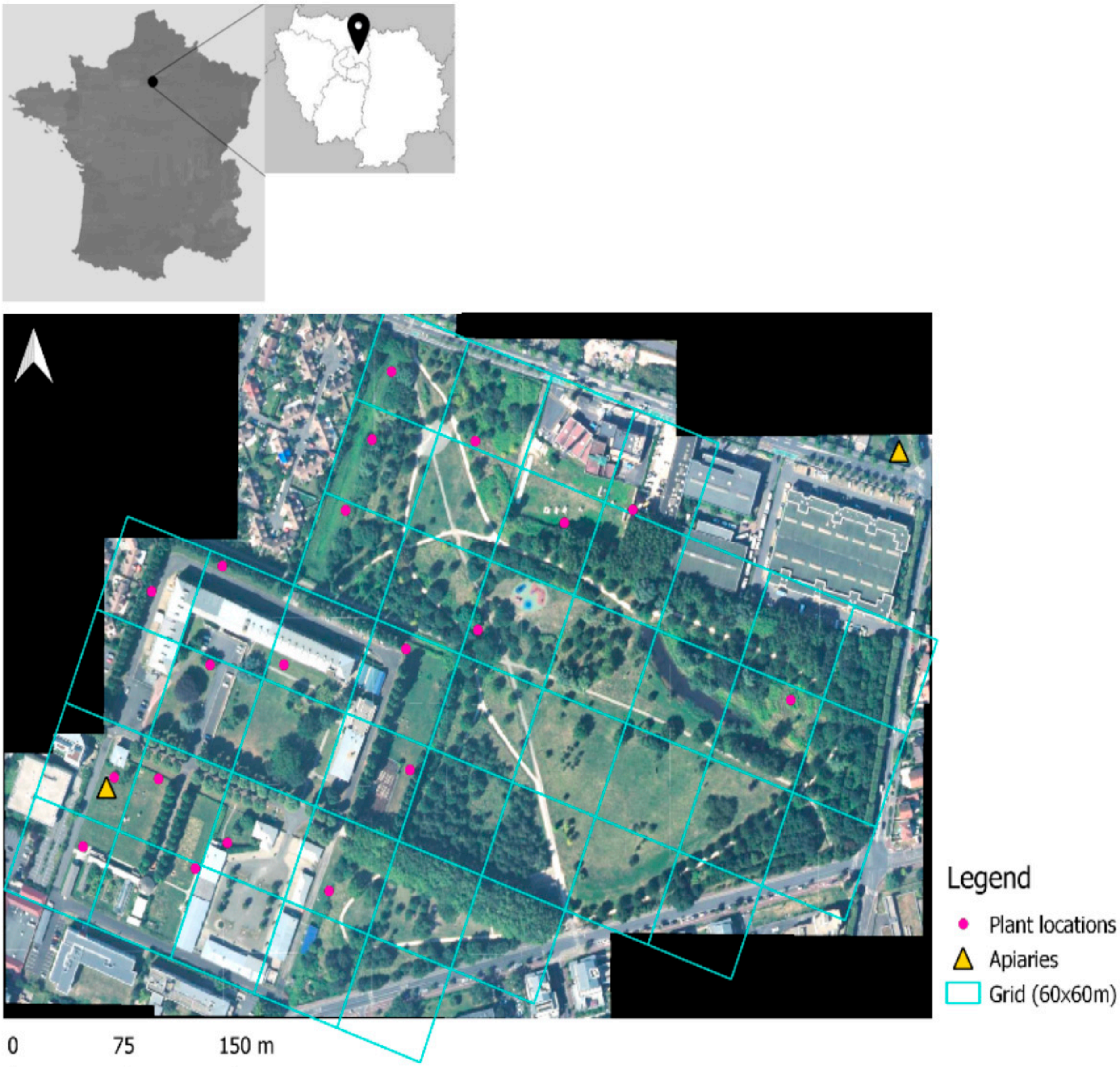

Figure S2. (a) Strawberry plants after potting, (b) example of two strawberry plants at their designated location with pollinator exclusion bags around the open flowers, (c) a strawberry flower excluded from insect visits by means of a pollinator exclusion bag, (d) a Diptera visiting a strawberry flower.

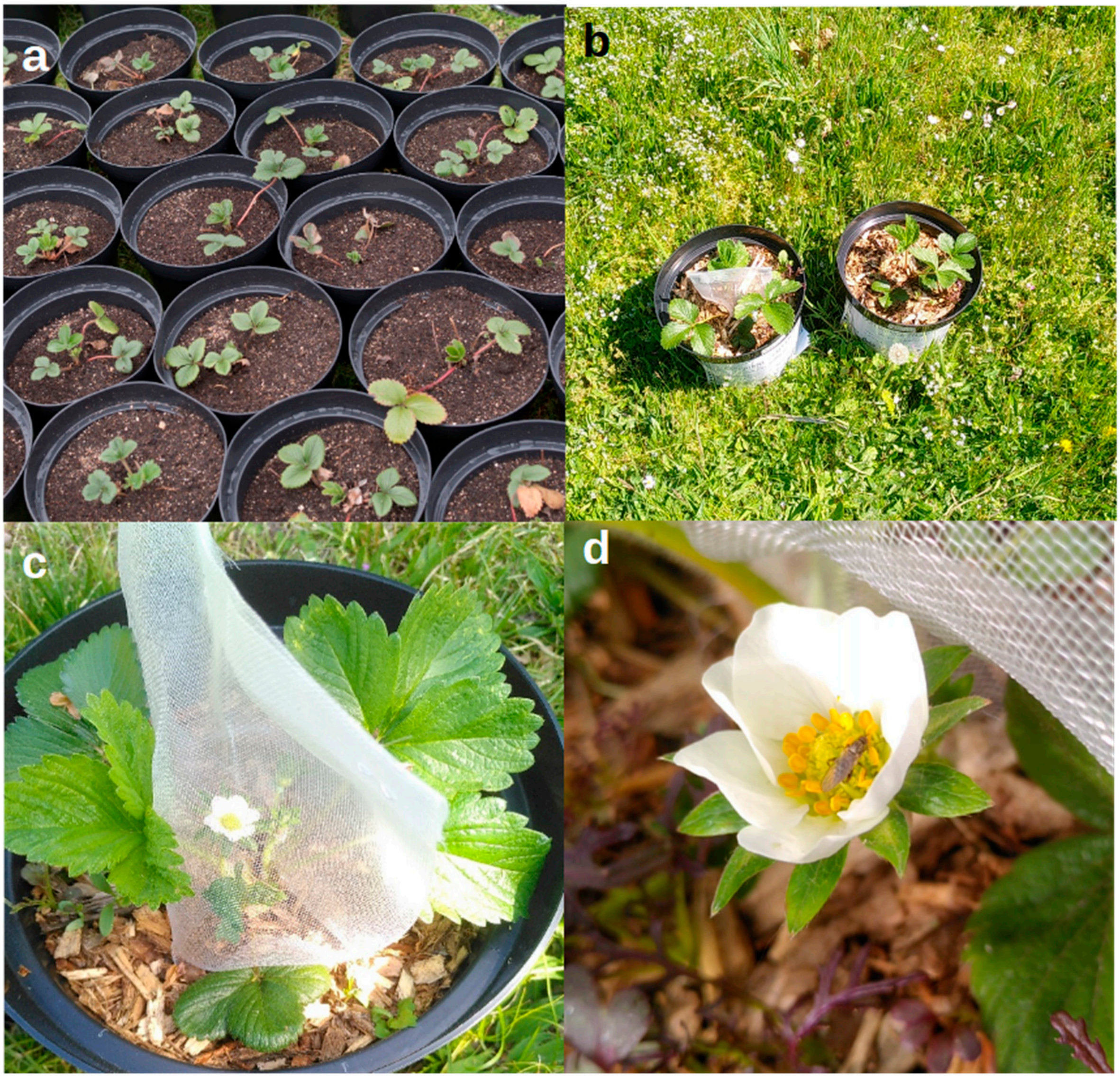

Figure S3. Example of a strawberry from the experiment showing a malformation.

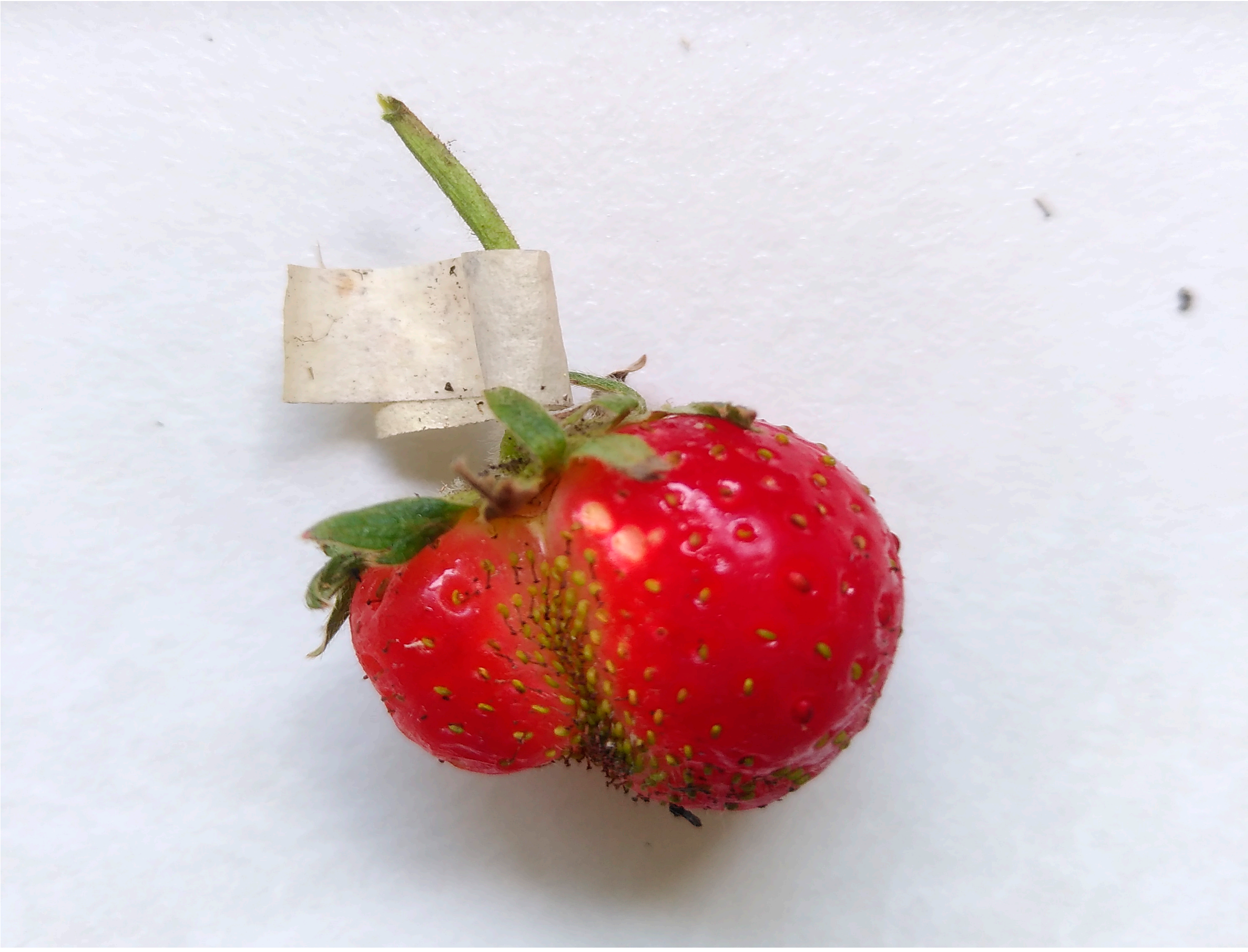

Supplement: Supplementary file 1 [file insects-14-00877-s001.zip › insects-2654830-supplementary.pdf]
